# Altered Clock Gene Expression in Obese Visceral Adipose Tissue Is Associated with Metabolic Syndrome

**DOI:** 10.1371/journal.pone.0111678

**Published:** 2014-11-03

**Authors:** Elaine Vieira, Elena G. Ruano, Ana Lucia C. Figueroa, Gloria Aranda, Dulce Momblan, Francesc Carmona, Ramon Gomis, Josep Vidal, Felicia A. Hanzu

**Affiliations:** 1 CIBER de Diabetes y Enfermedades Metabólicas Asociadas (CIBERDEM), Barcelona, Spain; 2 Diabetes and Obesity Research Laboratory, IDIBAPS, Barcelona, Spain; 3 Department of Endocrinology and Nutrition, Hospital Clinic, Barcelona, Spain; 4 Institute Clinic of Gynecology, Obstetrics and Neonatology, Hospital Clinic, Universitat de Barcelona, Barcelona, Spain; 5 Department of Gastrointestinal Surgery, Hospital Clinic, Universitat de Barcelona, Barcelona, Spain; 6 Faculty of Medicine, University of Barcelona, Barcelona, Spain; University of Lübeck, Germany

## Abstract

Clock gene expression was associated with different components of metabolic syndrome (MS) in human adipose tissue. However, no study has been done to compare the expression of clock genes in visceral adipose tissue (VAT) from lean and obese subjects and its clinical implications. Therefore, we studied in lean and obese women the endogenous 24 h expression of clock genes in isolated adipocytes and its association with MS components. VAT was obtained from lean (BMI 21–25 kg/m^2^; n = 21) and morbidly obese women (BMI >40 kg/m^2^; n = 28). The 24 h pattern of clock genes was analyzed every 6 hours using RT-PCR. Correlation of clinical data was studied by Spearman analysis. The 24 h pattern of clock genes showed that obesity alters the expression of *CLOCK, BMAL1, PER1, CRY2* and *REV-ERB ALPHA* in adipocytes with changes found in *CRY2* and *REV-ERB ALPHA* throughout the 24 h period. The same results were confirmed in VAT and stromal cells (SC) showing an upregulation of *CRY2* and *REV-ERB ALPHA* from obese women. A positive correlation was observed for *REV-ERB ALPHA* gene expression with BMI and waist circumference in the obese population. Expression of *ROR ALPHA* was correlated with HDL levels and *CLOCK* with LDL. Obese subjects with MS exhibited positive correlation in the *PER2* gene with LDL cholesterol, whereas *REV-ERB ALPHA* was correlated with waist circumference. We identified *CRY2* and *REV-ERB ALPHA* as the clock genes upregulated in obesity during the 24 h period and that *REV-ERB ALPHA* is an important gene associated with MS.

## Introduction

Circadian clocks regulate 24 hours rhythms in physiology and behavior that are essential to maintaining normal metabolic function. In humans, disruption of circadian rhythms (e.g., shift work) is associated with increased adiposity and increased risk of obesity and type 2 diabetes [1], [2]. Moreover, recent studies have linked polymorphisms of human circadian clock genes with metabolic dysfunction [3], [4]. Adipose tissue is a metabolic active organ that exhibits a rhythmic behavior [5]. In a single time point study, clock gene expression was associated with different components of metabolic syndrome in human adipose tissue [6]. Thus, it is of extreme importance to identify which clock genes are disrupted in obesity for an optimization of clinical interventions aimed at reducing the incidence of metabolic diseases such as type 2 diabetes.

The rhythmic expression of circadian clocks exists not only in the master clock in the brain but also in other tissues [7]. Clock genes were shown to oscillate independently of the suprachiasmatic nucleus exhibiting different circadian oscillations between subcutaneous and visceral adipose explants in morbidly obese women [8]. Intriguingly, in subcutaneous adipose tissue taken from lean, obese and type 2 diabetes subjects, the circadian expression of clock genes was comparable among the groups [9]. To date, there are no studies comparing the 24 h expression of clock genes in human visceral adipose tissue (VAT) from lean and obese subjects as well as the expression of clock genes in different cell fractions of human VAT.

In this study, we examined the 24 h expression profile of clock genes in adipocytes from lean and morbidly obese women. We identified *CRY2* and *REV-ERB ALPHA* as the clock genes upregulated in obesity during the 24 h period. The upregulation of these genes was also confirmed in VAT and in the stromal cells from lean and obese women. In this context, we found a positive correlation of *REV-ERB ALPHA* expression levels with metabolic syndrome parameters.

## Materials and Methods

### Subjects

Twenty-one lean, drug-free, non-obese healthy women (BMI 23.18±1.8 kg/m^2^, age 42.95±6.6) undergoing elective procedures of gynecological surgery to correct benign conditions (miomas or vaginal prolapsed) and twenty-eight morbidly obese woman (BMI 45.37±4.3 kg/m^2^, age 46.07±11.3) undergoing bariatric surgery at the Obesity Unit of the Hospital Clinic of Barcelona were included in the study. The study was approved by the Ethics Committee of the Hospital Clinic of Barcelona, Spain. Written informed consent was obtained from all participants. Clinical history and physical data were registered.

In both groups, participants with a diagnosis of diabetes [10] and on glucose-lowering or insulin-sensitizer medication or insulin or on diets were excluded. The exclusion of diabetic patients have been mainly due to the use of metformin since AMP activated protein kinase (AMPK) activators were shown to regulate the pattern of clock genes [11], [12], [13], [14]. Subjects receiving hormones (i.e. glucocorticoids, oral contraceptives, or other substitutive hormonal treatment) or medications known to affect metabolism, or with a recent history of malignant disease or a major chronic disease, smokers and subjects with sleep disorders or shift work were also excluded from both groups. Main biochemical parameters (Table 1) were measured in serum, after overnight fasting in the laboratory of our hospital using standard assays. Metabolic syndrome components were defined using International Diabetes Federation (IDF) criteria (www. idf.org).

**Table 1:** Clinical characteristics of the population.

	LEAN	OBESE		
		TOTAL	MS	NON MS
Number	21	28	10	18
Age (years)	42.95±6.6	46.07±11.3	49.80±12.2	44.0±10.6
BMI	23.18±1.8	45.37±4.3***	45.44±4.6	45.33±4.32
Waist circumference (cm)	ND	126.50±10.4	125.38±8.5	127.0±11.3
Fasting glucose (mmol/l)	4.84±0.49	5.55±0.44*	5.79±0.55†	5.42±0.30
HbA1c % - (NGPS/DCC)	ND	5.73±0.27	5.78±0.2	5.70±0.3
Total Cholesterol (mmol/l)	4.89±1.05	5.06±0.90	5.02±0.80	5.10±0.94
LDL (mmol/l)	3.49±0.2	3.84±0.71	3.85±0.64	3.86±0.83
HDL (mmol/l)	1.25±0.21	1.16±0.39*	1.14±0.59	1.18±0.25
Triglycerides (mmol/l)	1.11±0.10	1.52±0.07	1.89±0.94	1.36±0.37
SBP (mm Hg)	124.3±7	127.33±9.7	130.11±10.1	125.67±9.4
DBP (mm Hg)	71.5±3.9	78.66±8.6	80.0±7.5	77.87±9.4

Data are mean ±SEM.

*p<0.05;

***p<0.001 lean vs. obese group.

† p<0.05 MS vs. NON MS group. MS =  Metabolic Syndrome. SBP =  systolic blood pressure and DBP = diastolic blood pressure.

### Adipose tissue collection and isolation of adipocytes and stromal cells

Visceral (omental) adipose tissue biopsies from the morbidly obese (BMI 45.37±4.3 kg/m^2^, n = 28) and non-obese individuals (BMI 23.18±1.8 kg/m^2^, n = 21) were obtained during laparoscopic surgery at 8:00 am at the Hospital Clinic, Barcelona, Spain. The tissue was then immediately transported to the laboratory for the experimental studies. Adipose tissue was thereafter carefully dissected from skin and vessels under sterile conditions. The VAT was then divided in two parts: one small piece of whole VAT was rapidly frozen at −80°C for the measurements of gene expression. The other piece was used for isolation of adipocytes and stromal cells. Briefly, VAT was cut in small pieces and incubated at 37°C for 30 to 60 min in a collagenase solution (4 ml/1 g of tissue, Sigma, St Louis) to isolate adipocytes and stromal cells. After digestion, the adipose tissue was washed with PBS and centrifuged for 5 min at 25°C at 400 g in order to separate the different fractions of cells. The adipocytes and stromal cells were separated into different tubes and the stromal cells were treated with an erythrocyte lysis buffer for 5 min. They were then washed 2 times with PBS and centrifuged 2 times for 5 min each at 25°C at 400 g, and rapidly frozen at −80°C. The adipocytes were then washed 2 times with PBS and centrifuged 2 times for 5 min each at 25°C at 400 g for collagenase clearance. After washing, the adipocytes were divided into 5 different petri dishes named T0, T6, T12, T18 and T24 and cultured in M199 culture medium (Sigma, St Louis) for 8 h in order to allow the adipocytes to adapt to the new environmental conditions in incubator with a humidified atmosphere containing 5% CO_2_. After adaptation, adipocytes were synchronized with a serum-shock for 2 hours, a standard method to study clock genes in vitro in several cell types [15]. After synchronization, adipocytes were collected every 6 hours during a 24 h period for gene expression analysis. Due to small amount of stromal cells, we could not perform the 24 h expression pattern of clock genes in this cell type. In addition, the 24 h expression pattern of clock genes in whole VAT was not performed due to the presence of stromal cells in this tissue that might contribute to the 24 h expression pattern of clock genes and thus interfering in the results.

### RNA extraction and gene expression

Total RNA was isolated from whole VAT and isolated adipocytes using a RNeasy Lipid Tissue Mini Kit (QIAGEN, Hilden, Germany). Total RNA from stromal cells was isolated using a RNeasy Mini kit Plus (QIAGEN, Hilden, Germany). RNA was quantified using a Nanodrop 1000 (Thermo Scientific, Wilmington, MA) and it was reverse transcribed using a High Capacity cDNA Reverse Transcription Kit (AB Applied Biosystems, USA) following the manufacturer's instructions. Real time PCR was carried out in a Light Cycler 480 System (Roche, Basel, Switzerland) using MESA GREEN qPCR MasterMix Plus for SYBR Assay (Eurogentec, Liège, Belgium). The housekeeping genes *RPLP0* (ribosomal protein large P0, *alias 36B4*) and *ACTIN* was used as the arrhythmic endogenous control for quantification [16], [17], [18]. Values were expressed as the relative expression respective to control levels (2^−ΔΔct^). The primer sequences are shown in Table 2.

**Table 2:** Quantitative real-time PCR primers.

NAME	Sense Primer (5′-3′)	Antisense primer (5′-3′)
*BMAL1*	CTGGCTAGAGTGTATACGTTTGG	GGTCACCTCAAAGCGATTTTC
*CLOCK*	AAAATACTCTCTACTCATCTGCTGG	ATGGCTCCTTTGGGTCTATTG
*PER1*	ACATGTCCACCTATACCCTGG	CCTGCTCCGAAATGTAGACG
*PER2*	GCCAGAGTCCAGATACCTTTAG	TGTGTCCACTTTCGAAGACTG
*CRY1*	TTACACTATGCTCATGGCGAC	GTGCTCTGTCTCTGGACTTTAG
*CRY2*	CTCTGTCTACTGGCATCTGTC	GCTTCCAGCTTGCGTTTG
*RORA*	CTAGCTCTTCAACACGTCCTAC	TCGCACAATGTCTGGGTATATT
*REV-ERB ALPHA*	ACCAAGTCACCCTGCTTAAG	CATCACTGTCTGGTCCTTCAC
*PPAR GAMMA 2*	CCCATTCCTTCACTCATA	CTTCCATTACCCACACATCC
*NAMPT*	GAGACTGCCGGCATAGGGGC	GGTACTGTGCTCTGCCGCTGG
*36B4*	CGACCTGGAAGTCCAACTAC	ATCTGCTGCATCTGCTTG
*C/EBPA*	CGCTGCGGCCGCTGGTGAT	GCGGCGGCGGCTGGTAAGG
*C/EBPB*	CAGCCACCAGCCCCCTCACTA	GCGCGGCCTCCCTGCTCTG

### Statistical and Cosinor Analysis

Data is shown as mean ±SEM. Comparison among clock genes between lean and obese were analyzed using Student's *t* test. Gene expression time course using two-way ANOVA (factors time and group) was quantified with a level of significance of p<0.05. Association between phenotype data and clock gene expression was studied by Spearman correlation analysis. The Cosinor analysis (Table 3) was done by using the Acro software and include the mesor (middle value of the fitted cosine representing a rhythm adjusted mean), the amplitude (difference between the minimum and maximum of the fitted cosine function), and the acrophase (the time at which the peak of a rhythm occurs, expressed in hours).

**Table 3:** Cosinor Analysis of the 24 h expression pattern of Clock genes.

GENE	MESOR			AMPLITUDE			ACROPHASE		
	LEAN	OBESE	pVALUE	LEAN	OBESE	pVALUE	LEAN	OBESE	pVALUE
*CLOCK*	0.70	0.77	0.61	0.35	0.25	0.46	14.4 h	8.6 h	0.16
*BMAL1*	0.65	0.84	0.14	0.36	0.40	0.72	11.7 h	9.8 h	0.65
*PER1*	0.63	0.52	0.42	0.33	0.37	0.77	11.7 h	5.4 h	0.07
*PER2*	0.67	0.55	0.44	0.31	0.37	0.65	5.7 h	2.9 h	0.14
*CRY1*	0.47	0.41	0.57	0.39	0.26	0.15	3.4 h	6.2 h	0.20
*CRY2*	0.55	1.1	0.0001***	0.34	0.64	0.001**	5.7 h	3.1 h	0.27
*REV ERBA*	3.7	10.9	0.001**	3.3	7.4	0.003**	15.5 h	15.8 h	0.81
*RORA*	0.61	0.72	0.51	0.31	0.30	0.80	9.7 h	8.9 h	0.85

Data are mean ±SEM.

**p<0.001.

***p<0.001 lean vs. obese group.

## Results

### Clinical characteristics of the population

The clinical characteristics of the population studied are presented in Table 1. The lean and the obese groups did not statistically differ with respect to age. The obese population had a BMI greater than 40 kg/m^2^ indicating morbid obesity. Mean fasting glucose was significantly higher in the obese group as compared with lean subjects, whereas HDL– cholesterol was lower in the obese group. Total and LDL - cholesterol and triglycerides were higher (p = 0.06), although not at statistically significant levels, in obese subjects, while systolic and diastolic blood pressure did not statistically differ between lean and obese groups. Among the morbidly obese subjects, 35.7% (10 out of 28) fulfilled the metabolic syndrome criteria defined by the IDF panel. Fasting glucose was higher in obese subjects with MS as compared to non MS obese subjects. Regarding comorbidities, 50% (14 of 28) presented hypertension and were on treatment with antihypertensives drugs (hydrochlorothiazide and calcium blockers); 32% (9 of 28) presented untreated dyslipidemia; and 28.7% (8 out of 28) of the obese patients presented impaired fasting glucose levels.

### The 24 h expression of clock genes and metabolic genes in isolated adipocytes from lean and obese subjects

**Figure 1:**
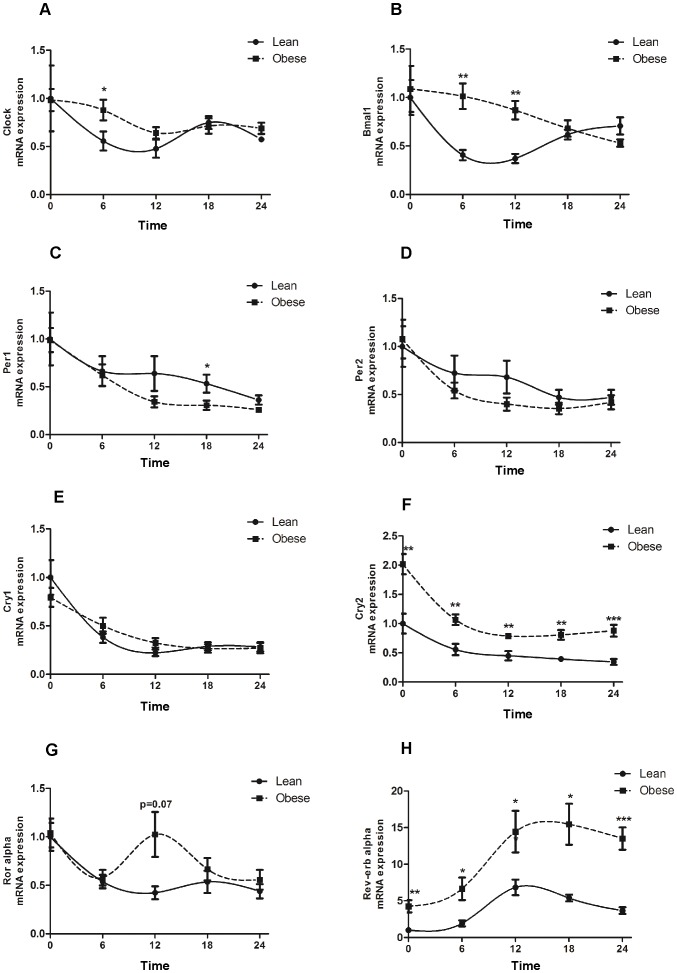
24 h of clock gene expression profiles in human adipocytes from lean and obese subjects. Circadian clock gene expression in adipocytes from lean (full line) and obese (dashed line) subjects. (**A**) *CLOCK* gene expression. (**B**) *BMAL1* gene expression. (**C**) *PER1* gene expression (**D**) *PER2* gene expression (**E**) *CRY1* gene expression (**F**) *CRY2* gene expression (**G**) *ROR ALPHA* gene expression. (**H**) *REV-ERB ALPHA* gene expression. *p<0.05; **p<0.01; ***p<0.001; Data are expressed as mean ±S.E.M of n = 10–15. The relative expression of clock genes is calculated and normalized by *36B4* house keeping gene. Time =  Time in culture after serum shock treatment.

The 24 hour measurements showed that *CLOCK* and *BMAL1* were upregulated in adipocytes from obese subjects at different time (T) points: *CLOCK* at T6 (Fig. 1A) and *BMAL1* at T6 and T12 (Fig. 1B) whereas *PER1* was downregulated at T18 (Fig. 1C). No changes were found in *PER2* (Fig. 1D), *CRY1* (Fig. 1E) or *ROR ALPHA* (Fig. 1G) gene expression between lean and obese subjects. Interestingly, *CRY2* (Fig. 1F) and *REV-ERB ALPHA* (Fig. 1H) expression exhibited increased gene expression over the course of 24 hours in the obese group. These results demonstrated that *CRY2* and *REV-ERB ALPHA* are the clock genes that are altered over the course of 24 hours in adipocytes from obese women. Table 3 shows the cosinor analysis of the 24 h expression pattern of clock genes. Among the clock genes studied only *REV-ERB ALPHA* and *CRY2* showed statistical significance in the mesor and amplitude. The mean amplitude and mesor of *REV-ERB ALPHA* expression was increased in obese subjects as compared to lean subjects (p = 0.003; p = 0.001, respectively). The same results were found for *CRY2* with a mesor (p = 0.0001) and amplitude (p = 0.001) increased in the obese subjects as compared to lean subjects.

**Figure 2:**
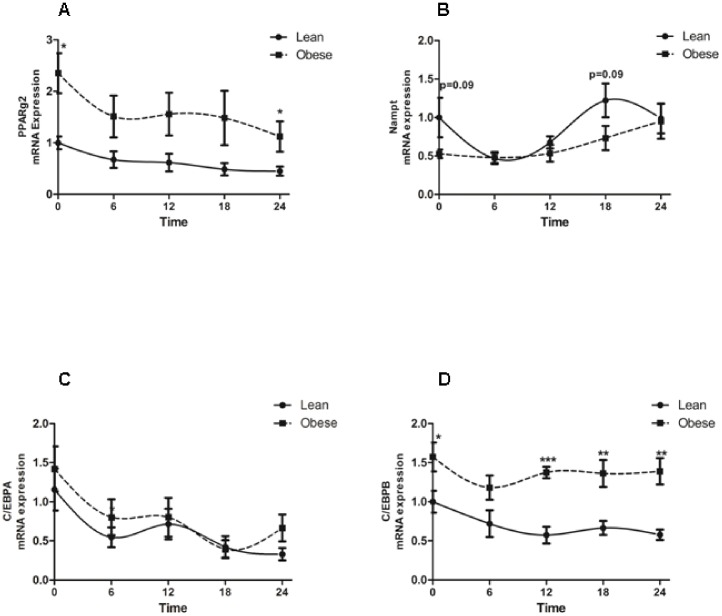
24 h expression profiles of metabolic genes in human adipocytes from lean and obese subjects. Circadian clock gene expression in adipocytes from lean (full line) and obese (dashed line) subjects. (**A**) 24 h expression pattern of *PPARG2* gene (**B**) 24 h expression pattern of *NAMPT* gene expression. (**C**) 24 h expression pattern of *C/EBPA* gene (**D**) 24 h expression pattern of *C/EBPB* gene. *p<0.05; **p<0.01; ***p<0.001. Data are expressed as mean ±S.E.M of n = 9–13; values are normalized by *36B4* house keeping gene. Time =  Time in culture after serum shock treatment.

A large body of evidence from human and animal studies has demonstrated that the regulation of molecular clocks is linked to pathways of energy metabolism. Therefore, we next examined the mRNA expression of metabolic genes known to regulate adipocyte cell metabolism and clock genes. We measured the 24 h mRNA levels of peroxisome proliferator-activated receptor gamma 2 (*PPARG2*) and nicotinamide phosphoribosyltransferase (*NAMPT*; the rate-limiting enzyme in NAD^+^ biosynthesis), which were found to regulate clock genes [19]. The 24 h pattern of *PPARG2* expression showed increased expression levels at T0 and T24 in adipocytes from obese subjects as compared to lean subjects (Fig. 2A). The 24 h mRNA expression of *NAMPT* showed a strong statistically significant tendency (p = 0.09) to be reduced at T0 and T18 in adipocytes from obese subjects (Fig. 2B). In addition, we measured the expression of CCAAT/enhancer binding protein alpha (*C/EBPA*) and (*C/EBPB*), keys transcription factors involved in adipogenesis. The 24 h mRNA expression of *C/EBPA* was not different from lean and obese subjects (Fig. 2C). However, *C/EBPB* was upregulated at T0, T12, T18 and T24 in obese subjects as compared to lean subjects (Fig.2D). In whole VAT the expression of *PPARG2* was increased whereas the expression of *NAMPT* was decreased in obese subjects as compared to lean subjects (Figure S1 A and S1B, respectively). There was no changes in the mRNA expression of peroxisome proliferator-activated receptor alpha (*PPARALPHA*), gamma1 (*PPARG1*) and Sirtuin1 (*SIRT1*) between obese and lean subjects (Figure S1 C, D and E, respectively).

Thus, obesity alters the 24 h expression pattern of clock genes and metabolic genes in human adipocytes. Furthermore, human adipocytes cultured in vitro retain some changes in the patterns of clock genes and metabolic genes during 24 h regardless of the contribution of the central clock located in the hypothalamus.

### Expression of clock genes in VAT and SC from lean and obese subjects

**Figure 3:**
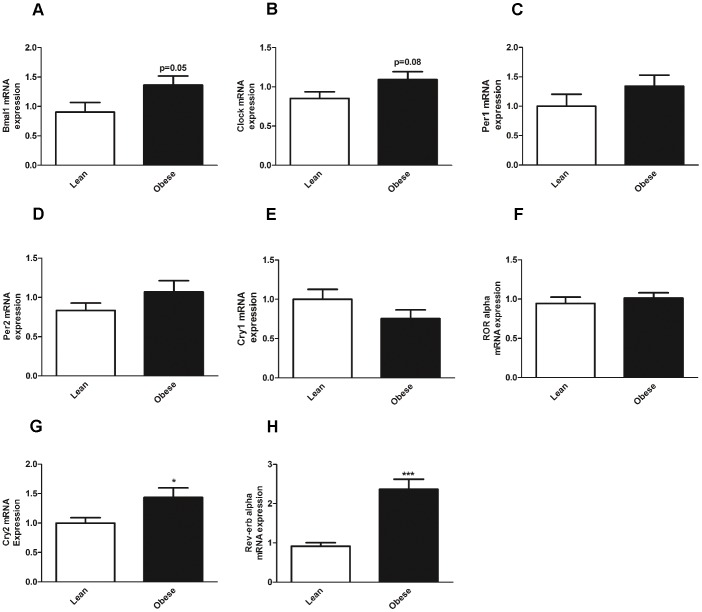
Clock gene expression in VAT from lean and obese subjects. Clock gene expression in human VAT from lean (white bars) and obese subjects (black bars). (**A**) *BMAL1* gene expression (**B**) *CLOCK* gene expression (**C**) *PER1* gene expression (**D**) *PER2* gene expression. (**E**) *CRY1* gene expression (**F**) *ROR ALPHA* gene expression (**G**) *CRY2* gene expression (**H**) *REV-ERB ALPHA* gene expression. *p<0.05; ***p<0.001. Data are expressed as mean ±S.E.M of n = 20–28. The relative expression of clock genes is calculated and normalized by *36B4* house keeping gene.

To confirm whether *CRY2* and *REV-ERB ALPHA* are altered in human obesity, we next measured the mRNA expression of clock genes in VAT and stromal cells from lean and obese subjects. The expression pattern of the core clock gene *BMAL1* had a tendency for upregulation in VAT from obese subjects (p = 0.05) (Fig. 3A). There were no statistical differences in gene expression in *CLOCK* (Fig. 3B), *PER1* (Fig. 3C), *PER2* (Fig. 3D), *CRY1* (Fig. 3E) and *ROR ALPHA* (Fig. 3F). Strikingly, *CRY2* and *REV-ERB ALPHA* were upregulated in VAT from obese as compared to VAT from lean subjects (Fig. 3G and 3H), respectively. Interestingly, among the clock genes studied, *REV-ERB ALPHA* exhibited the highest expression levels in obese subjects as compared to lean subjects. These results indicate that obesity can disrupt the expression of clock genes in human VAT, and that this disruption can be clock gene-specific, such as in the case of *CRY2* and *REV-ERB ALPHA*.

**Figure 4:**
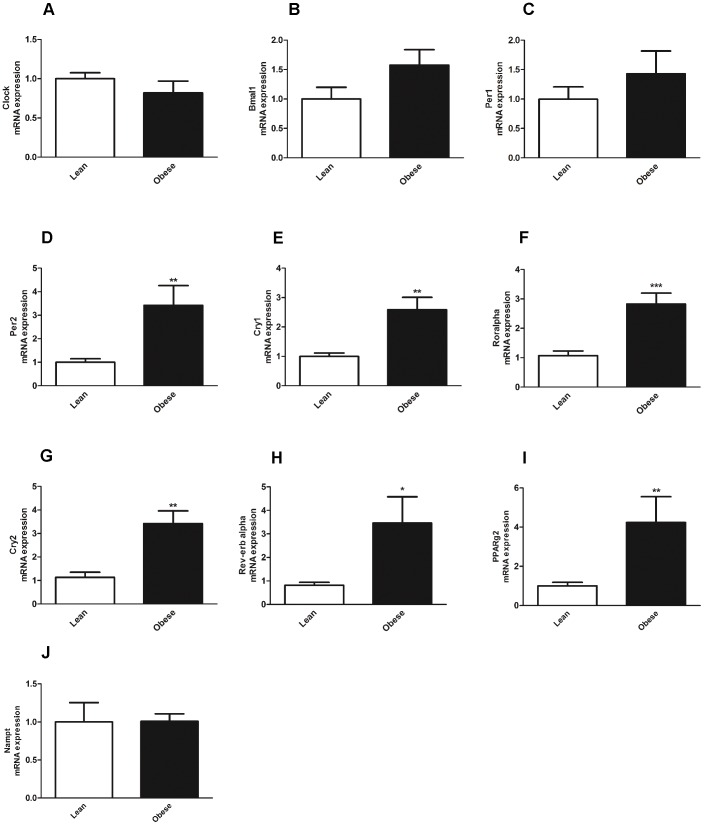
Clock gene expression in SC from lean and obese subjects. Clock gene expression in human SC from lean (white bars) and obese (black bars) subjects. (**A**) *CLOCK* gene expression (**B**) *BMAL1* expression (**C**) *PER1* gene expression (**D**) *PER2* gene expression (**E**) *CRY1* gene expression (**F**) *ROR ALPHA* expression gene expression (**G**) *CRY2* gene expression (**H**) *REV-ERB ALPHA* gene expression (**I**) *PPARG2* gene expression (**J**) *NAMPT* gene expression. *p<0.05; **p<0.01. Data are expressed as mean ±S.E.M of n = 10–28; values were normalized by *36B4* house keeping gene.

In SC we found no statistical difference in the expression levels of *CLOCK* (Fig. 4A), *BMAL1* (Fig. 4B), or *PER1* (Fig. 4C), between lean and obese subjects. There was an increase of gene expression of *PER2* (Fig. 4D), *CRY1* (Fig. 4E) and *ROR ALPHA* (Fig. 4F) in the obese as compared to lean subjects. Interestingly, *CRY2* (Fig. 4G) and *REV-ERB ALPHA* (Fig. 4H) were also found to be upregulated in stromal cells from obese as compared to lean subjects. *PPARG2* expression levels were increased in SC from obese subjects (Fig. 4I) whereas *NAMPT* gene expression levels were similar (Fig. 4J) in both groups. These results indicate that the changes in *PER2*, *CRY1* and *ROR ALPHA* from obese subjects were unique for the stromal cells fraction, whereas changes in *CRY2* and *REV-ERB ALPHA* occurred in adipocytes, stromal cells and VAT. Thus, our results identified *CRY2* and *REV-ERB ALPHA* as the clock genes that are altered in all cell fractions in the visceral adipose tissue from obese subjects.

### Correlation between clock gene expression in VAT and metabolic parameters

**Figure 5:**
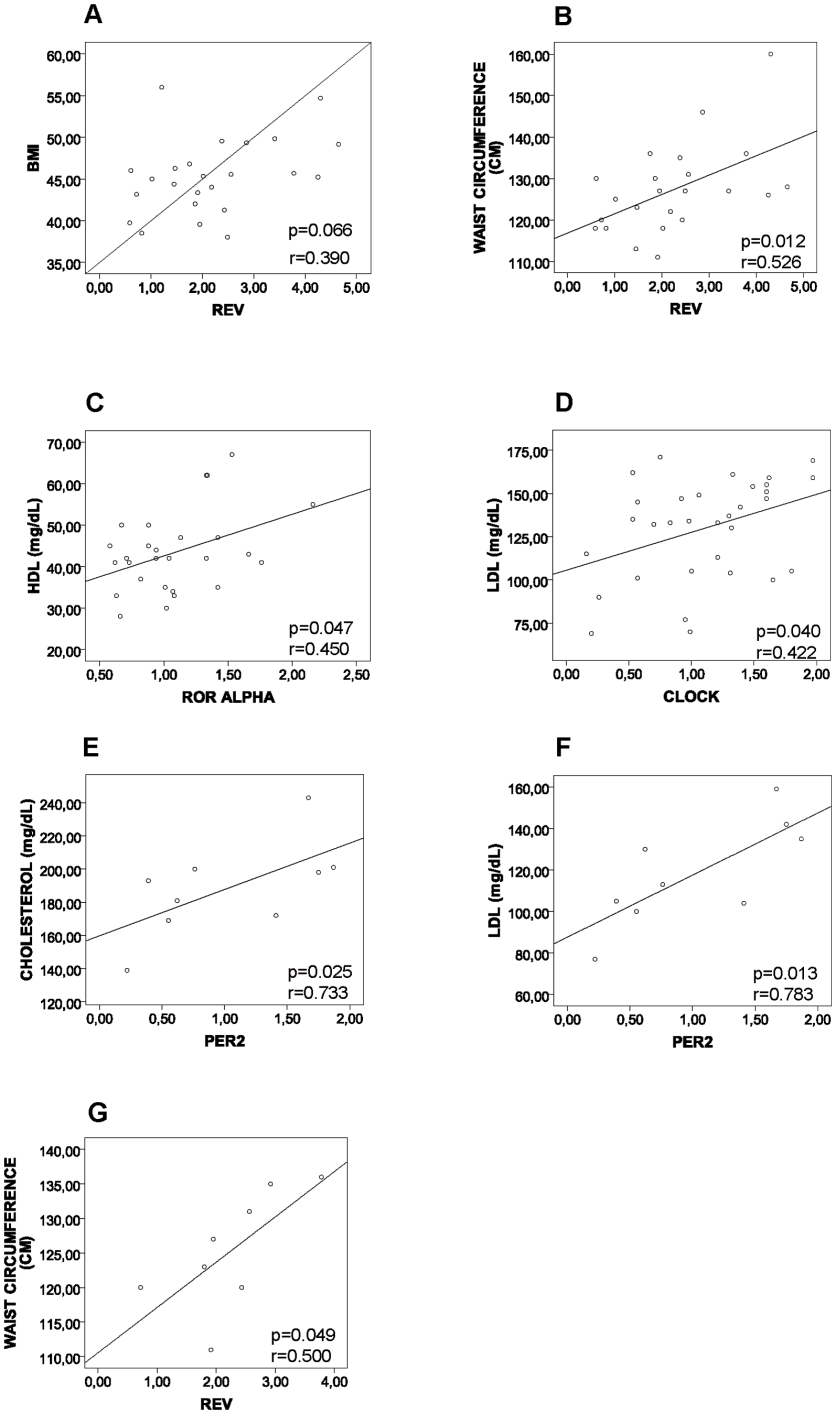
Correlation between clock genes and metabolic parameters. (**A**) *REV-ERB ALPHA* expression and BMI. (**B**) *REV-ERB ALPHA* expression and waist circumference (**C**) *ROR ALPHA* expression and HDL levels **(D**) *CLOCK* expression and LDL levels. (**E**) *PER2* expression and cholesterol levels in the MS group (**F**) *PER2* expression and LDL levels in the MS group. (**G**) *REV-ERB ALPHA* expression and waist circumference in the MS group. r =  Spearman correlation coefficient.

Associations between clock gene expression and several parameters of the clinical data obtained from the lean and obese groups were studied by Spearman correlation analysis and it was adjusted for age and BMI. When we analysed the whole population (lean and obese), we found a positive correlation of *REV-ERB ALPHA* gene expression in VAT with BMI (r = 0.552; p = 0.008, data not shown). In the obese population, among the clock genes studied, only *REV-ERB ALPHA* displayed a strong tendency for a positive correlation with BMI (r = 0.390; p = 0.066 and a statistically significant positive correlation with waist circumference (Fig. 5A and B). Possible correlations between clock gene expression and different cardiovascular risk factors were also analyzed. Overall, our results showed that *ROR ALPHA* (Fig. 5C) exhibited a positive correlation with HDL levels, whereas *CLOCK* (Fig. 5D) was correlated with LDL levels. We next refined our analysis of the obese population and separated it in two groups: one that did not have features of MS and was consequently named NON-MS, and one that presented the common features of MS according to IDF criteria. In the MS group, there was a positive correlation of *PER2* gene with total and LDL- cholesterol levels (Fig. 5E and F), respectively. Interestingly, *REV-ERB ALPHA* (Fig. 5G) gene expression was again positively correlated with waist circumference in the MS group. The correlations between *REV-ERB ALPHA* with BMI and waist circumference suggest that this nuclear receptor could have a potential role in obesity and metabolic syndrome.

## Discussion

This study provides the first evidence of altered clock gene expression in visceral adipose from obese subjects. In addition, we identified *REV-ERB ALPHA* as a clock gene that is upregulated in obesity and is positively correlated with BMI and waist circumference, key predictors of metabolic syndrome. Consistent with previous studies in VAT, our results confirm endogenous 24 h changes of clock genes expression in human VAT from obese women [8], [20]. However, our results differ from these studies in many ways. First, while the previous works studied Bmal1, Per 2 and Cry1 only in obese subjects, we included a broader range of clock genes in both obese and lean subjects. To our knowledge, for the first time, the present work provides data regarding endogenous changes of clock genes expression in human VAT from lean women. In addition, we used isolated adipocytes to address the intrinsic pattern of clock genes without the contribution of stromal cells. Thus, the robust oscillations of Bmal1, Per 2 and Cry1 in human adipose explants [8], [20] could come from the contribution of stromal cells. The present study did not include subcutaneous adipose tissue due to the fact that subcutaneous adipose tissue showed no difference in the circadian clock gene expression among lean, overweight and type 2 diabetic subjects [9].

Our data highlighted endogenous clock genes expression changes during 24 h in adipocytes that are independent of the central clock found in the brain, demonstrating impaired clock gene expression in VAT adipocytes from morbidly obese women. The absence of oscillations in some genes suggested that the central clock could be essential for coordination of the 24 h cycle in specific clock genes in human visceral adipose tissue. The changes in *CLOCK*, *BMAL1* and *PER1* expression occurred at specific time points with *BMAL1* exhibiting opposite changes in the expression pattern comparing with *REV-ERB ALPHA* expression in adipocytes from lean subjects. The lack of inhibitory effect of *REV-ERB ALPHA* on *BMAL1* expression in adipocytes from obese subjects might be due to impairment in the regulation of both genes in obesity. Interestingly, in whole fat tissue of mice with high fat induced obesity both Clock and Bmal1 genes presented a different tendency with a significant attenuated expression profile during 24 h while Per2 expression diminish only selective during the night period [21], while CLOCK ^Δ19^ and BMAL1(^−^/^−^) clock disruption determines low levels of FFA and glycerol with accumulation of triglycerides and increased adiposity [22].

One important finding of the present study was the upregulation of *CRY2* and *REV-ERB ALPHA* expression in the obese group throughout the 24 hour period. The role of *CRY2* and *REV-ERB ALPHA* in human adipose tissue is not known. In animal and cell lines studies cryptochromes (*CRY1* and *CRY2)*, critical interaction partners of Pers have been involved in lipid uptake [23]. When challenged with a hight fat diet Cry1/2 (^−^/^−^) mice rapidly gain weight despite displaying hypophagia and presented in adipose tissue an upregulated expression of lipogenic genes involved in insulin metabolism [23]. *REV-ERB ALPHA* is an important repressor of anti-adipogenic genes [24], [25] and recently polymorphisms in the *REV-ERB ALPHA* gene has been associated with obesity in different populations [26], [27], [28]. Indeed, *REV-ERB ALPHA* was shown to be a *PPARG* target gene, promoting the adipogenic activity of this gene in 3T3-L1 cells. Interestingly, our data showed an upregulation of *PPARG2* in isolated adipocytes at the baseline time point (T0) and again at 24 h (T24) and also in whole adipose tissue and stromal cells. This effect could not be explained by changes in *PER2* expression, since the Per2 gene was shown to control lipid metabolism by repressing PPARG in mice [29] and we found no difference in *PER2* expression between lean and obese during the 24 h period. However, the upregulation of *PPARG2* gene could also be explained by the increase in the expression of *C/EBPB* in the obese subjects. [30]. The downregulation of *NAMPT* in the obese group could also be linked with changes in the *REV-ERB ALPHA* gene, since this interaction has been observed in another cell type [17]. Although it is possible that the interactions among *REV-ERB ALPHA*, *PPARG2, C/EBPB* and *NAMPT* may also occur in human adipose tissue, this link should be investigated in future studies.

There has been growing evidence in recent literature of the role that circadian mechanisms play in adipose tissue biology, metabolism and obesity [6], [31], [32], [33]. Indeed, in the present work we found a positive correlation between clock genes and markers of metabolic syndrome, suggesting an important role of these genes in obesity and metabolic syndrome.

The relationship between *ROR ALPHA* and HDL levels found in our study is in agreement with what was found in a mice model with decreased and dysfunctional *ROR ALPHA* expression [34], [35]. These mice exhibit decreased adiposity and low HDL levels, suggesting that *ROR ALPHA* is a key modulator of fat accumulation and contributes to the susceptibility of these animals to developing atherosclerosis. The positive association between *CLOCK* and LDL levels in our study is in accordance with the link between the gene *CLOCK* and obesity in humans. Polymorphisms in the gene *CLOCK* have been associated with obesity parameters, such as cholesterol levels [36] and increased small dense LDL [37], in humans.

An important limitation of our study could be the low rate of metabolic syndrome in our population, as we have excluded from the study all patients on medications that might affect adipose tissue metabolism. Indeed, all obese patients with established type 2 diabetes and all being treated with oral antidiabetic agents such as metformin, have not been included in the study due to its effects on clock genes [11], [12], [13], [14]. Similarly, patients treated with statins have been also excluded. Despite a small number of subjects, our results in the MS group suggest a link between *PER2* and *REV-ERB ALPHA* genes with metabolic syndrome. Indeed, the associations between polymorphisms in the *PER2* gene locus may influence lipid metabolism by interacting with total serum fatty acids [38]. One very interesting finding of the present work is the upregulation of the *REV-ERB ALPHA* gene in all cells fractions over the course of 24 h in VAT and its correlation with BMI and waist circumference. It is worth noting that, even when we subdivided the obese group, the positive correlation of *REV-ERB ALPHA* and waist circumference was found in the MS group. In human subcutaneous adipose tissue, *REV-ERB ALPHA* has positively correlated with BMI in young obese subjects [39], and polymorphisms in this gene have been associated with obesity [26], [27], [28]. This is the first report regarding the association of *REV-ERB ALPHA* in human VAT with obesity and metabolic syndrome.

In summary, our data demonstrated that the expression pattern of clock genes *CRY2* and *REV-ERB ALPHA* is altered in VAT from morbidly obese subjects during the 24 h period as compared to lean subjects, with *REV-ERB ALPHA* identified as one of the most important clock genes associated with metabolic syndrome.

## Supporting Information

Figure S1 **Metabolic gene expression in VAT from lean and obese subjects.** Gene expression in human VAT from lean (white bars) and obese subjects (black bars). (**A**) *PPARG2* gene expression (**B**) *NAMPT* gene expression (**C**) *PPARALPHA* gene expression (**D**) *PPARG1* gene expression (**E**) *SIRT1* gene expression. **p<0.01. Data are expressed as mean ±S.E.M of n = 20–28; values were normalized by *36B4* house keeping gene.
(TIF)
