# Supplementary figures and images for: Altered Clock Gene Expression in Obese Visceral Adipose Tissue Is Associated with Metabolic Syndrome
Source: PLoS One. 2014 Nov 3;9(11):e111678. doi: 10.1371/journal.pone.0111678 (PMC4218799; doi:10.1371/journal.pone.0111678)

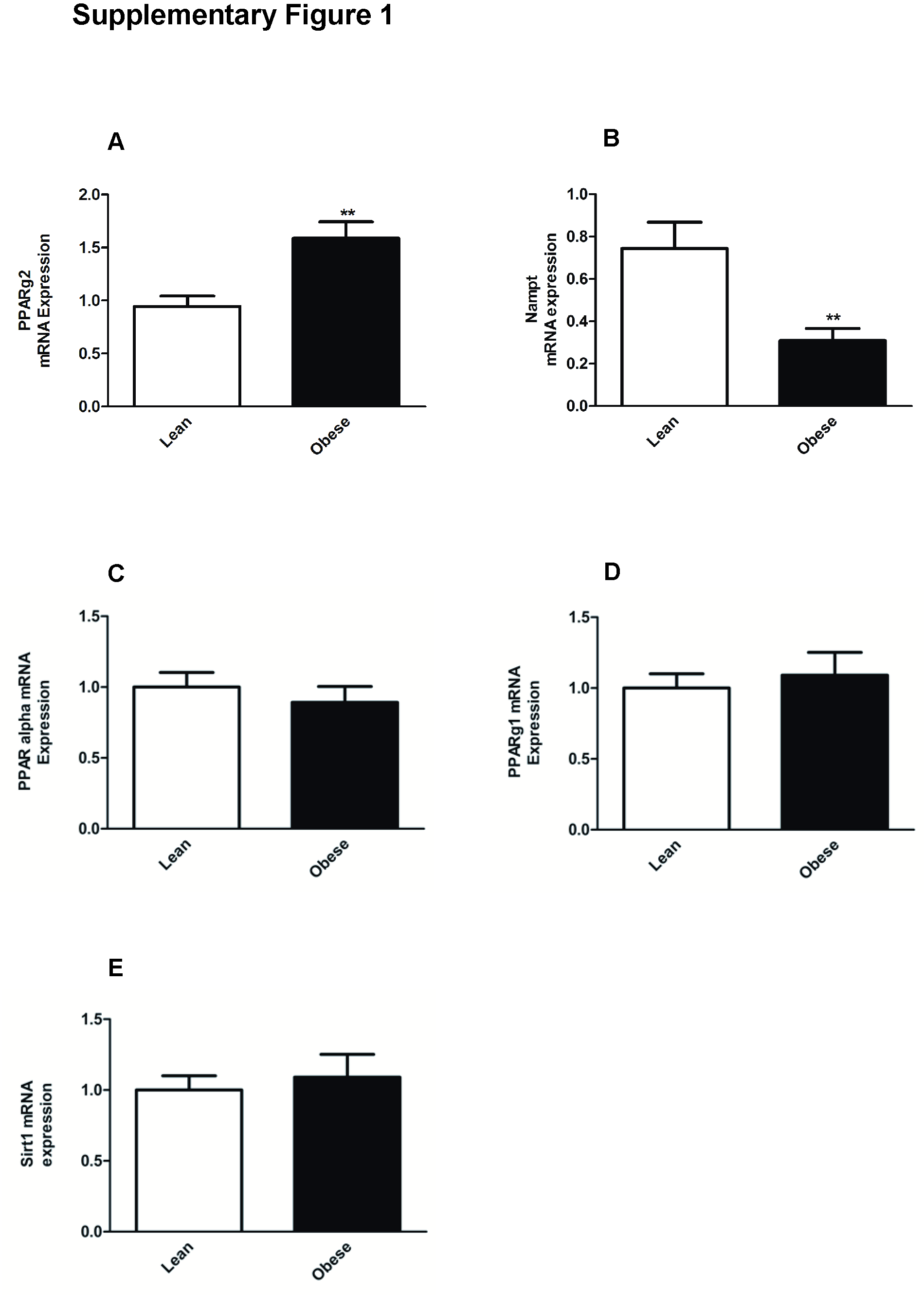

Supplement: Figure S1 — Metabolic gene expression in VAT from lean and obese subjects. Gene expression in human VAT from lean (white bars) and obese subjects (black bars). (A) PPARG2 gene expression (B) NAMPT gene expression (C) PPARALPHA gene expression (D) PPARG1 gene expression (E) SIRT1 gene expression. **p<0.01. Data are expressed as mean ±S.E.M of n = 20–28; values were normalized by 36B4 house keeping gene. (TIF) [file pone.0111678.s001.tif]
